# Supplementary material for: Acceptance and compliance with micronutrient powder (MNP) among children aged 6–23 months in northern Nigeria
Source: PLOS Glob Public Health. 2022 Oct 17;2(10):e0000961. doi: 10.1371/journal.pgph.0000961 (PMC10022258; doi:10.1371/journal.pgph.0000961)
Supplement: S1 Dataset — (ZIP) [file pgph.0000961.s008.zip › S8_Dataset/Direct Observations.zip!/Direct Observations/06072015_A_GA_AS_2_DO.pdf]

1.1.1 Instrument F –Direct Observation Form

<<**Instrument F Direct Observation Form**>>
**PI Name:** PragyaMathema,Stephen Kodish<br>**Study Title:** Formative research to develop an infant and young child feeding (IYCF) intervention with micronutrient powder (MNP) for children aged 6 – 23 months<br>**PI Version/Date:** Version1.0/January 24, 2015

Date: ___6/07/2015_____Location: _Sandoka community, Ganye____

Data Collector: Antiya Sanda__________Participant ID: A mud house near the forest ___

Main goals of this direct observation are to answer the following questions:

1. In this household, what would be the best way(s) to give nutritional supplements to young children? Why?

2. In this household, describe the food sharing that you observe throughout the meal. Are there any predictable patterns?

3. In this household, who is favored and how is this favoritism expressed during meal preparation and eating?

(TEAR OFF AT PERFORATED DOTTED LINE BELOW)

PARTICIPANT ID: _________

Household Composition Table (Fill out as you do data collection)

INITIALS	SEX (M/F)	AGE (estimated)	Relationship to household	Description
SB	M	9month	child	A small boy
ST	F	32yrs	mother	A slim tall woman
TG	F	16yrs	daughter	A teenage girl wearing blue wrapper
BS	F	8yrs	daughter	A black slim girl with a loosed hair
TB	M	35yrs	Father	A tall dark man wearing a black shirt and a trouser

PARTICIPANT ID: _________

TIME	ACTOR INITIALS	RECIPIENT INITIALS	WHAT HAPPENED	FOOD	Reactivity
11:oo	ST	SB	Breastfeeding	Breast milk	
11:o5	ST	SB	Breastfeeding	Breast milk	
11:10	ST	SB	Breastfeeding	Breast milk	
11:15	ST	SB	Breastfeeding	Breast milk	
11:20	ST	SB	Breastfeeding	Breast milk	
11:25	ST	SB	Breastfeeding	Breast milk	
11:30	SB		Sleeping		
11:35	SB		Sleeping		
11:40	SB		Sleeping		
11:45	SB		Sleeping		

TIME	ACTOR INITIALS	RECIPIENT INITIALS	WHAT HAPPENED	FOOD	Reactivity
11:50			Sleeping		
11:55	ST		Start making fire		
12:00	ST		Take a pot and fetch water	water	
12:05	ST		Put water on a fire	water	
12:10	ST		Bringing the cover of the pot to cover it	guinea corn	
12:15	ST		Washing her hands		
12:21	ST		Come out of the kitchen with guinea corn flour		
12:22	ST		Used her hand to mix the guinea corn flour with water		
12:23	ST		Mixing guinea corn and water using her hand	Guinea corn and water	
12:24	ST		Open the pot on fire to see if the water is boiling		
12:25	ST		Start pouring the mixed guinea corn flour into the pot	Guinea corn	
12:30	ST		Using the cup to stir the mix guinea corn flour on the fire		
12:35	ST		Bringing the pot down from the fire	guineacorn	
12:40	ST		Fetching the kunu inside a small cup	kunu	
12:45	ST		Start pouring water into the empty pot	water	
12:50	ST		In to the kitchen to bring spoon		
12:55	ST		Rinsing the spoon		R
12:56	ST		Keep the cup of the kunu on a mat	kunu	
12:57	ST		In to the room to bring the MNP	MNP	
12:58	ST		Keep the MNP on a mat	MNP	

TIME	ACTOR INITIALS	RECIPIENT INITIALS	WHAT HAPPENED	FOOD	Reactivity
12:59	ST		Carry the spoon of the kunu and start stirring it with the spoon to make it cold		
1:00	ST		Allow the kunu to get cold	kunu	
1:01	ST		Allow the kunu to get cold	kunu	
1:02	ST		Used the spoon to tested the kunu	kunu	
1:03	ST		Trying to unseal the MNP	MNP	
1:05	ST		Start pouring the MNP into the kunu	kunu	
1:06	ST		Throws the empty scathed of the MNP		
1:07	ST		Start mixing the MNP with the kunu	Kunu and MNP	
1:08	ST		Mixing the kunu with the MNP	Kunu and MNP	
1:09	ST		Mixing the kunu with the MNP		
1:10	ST		Check the cup to see if the MNP has blended with kunu		
1:12	ST		Use a spoon to taste the kunu mixed with MNP	Kunu and MNP	
1:13	ST		Feeding her child kunu using a small spoon	kunu	
1:15	ST	SB	The child is slowing	kunu	
1:16	ST	SB	Feeding her child	kunu	
1:17	ST	SB	Feeding her child	kunu	
1:18	ST	SB	Feeding her child	kunu	
1:19	ST	SB	Feeding her child	kunu	
1:20	ST	SB	Feeding her child	kunu	
1:25	ST	SB	Using water to clean the baby's		

TIME	ACTOR INITIALS	RECIPIENT INITIALS	WHAT HAPPENED	FOOD	Reactivity
			face		
1:30	ST	SB	Check the cup to see if the child has drink all the kunu mixed with MNP		
1:35	ST		Poured water into the cup of the kunu		
1:40	ST	SB	Lays her child on the mat		
1:45	ST		Take the empty cup to wash		
1:50	ST		Using a soap to wash the empty cup		R
1:55	ST	SB	Breast milk	Breast milk	
2:00	ST	SB	Breast milk	Breast milk	
2:05	ST	SB	Breast milk	Breast milk	
2:10	ST	SB	Breast milk	Breast milk	
2:15	ST	SB	Breast milk	Breast milk	
2:20	ST	SB	Breast milk	Breast milk	
2:25	TG		Gather dirty plates to wash		
2:30	TG		Start fetching water to rinse the plates		
2:35	TB		Washing dirty plates		
2:40	BS,TG		BS have come out to join the sister to rinse the plates		
2:45	BS,TG		Are washing the plate together		
2:50	TGBS,		Are washing the plates		
2:55	TG		tuber of Bring out a yam from the kitchen	yam	
3:00	TG		Fetched water into a big pot		

TIME	ACTOR INITIALS	RECIPIENT INITIALS	WHAT HAPPENED	FOOD	Reactivity
3:05	TG		Start peeling the yam with knife	yam	
3:10	TG		Peeling yam	yam	
3:15	TG		Start slicing the yam		
3:20	TG		Slicing yam		
3:25	TG		Empty the water from the yam and poured another water on the yam		
3:30	TG		Carry the pot of the yam and set it on firewood stand		
3:35	TG		Bring the cover of the pot		
3:40	TG		Add salt in to the yam		
3:45	TG		Start boiling on the fire		
3:50	TG		Sweeping the yam peels		
3:55	TG		Come to site on the mat		
4:00	ST	BS	Combing her daughter her		
4:05	ST	BS	Starts plaiting her child		
4:10	ST	BS	plaiting her child		
4:15	ST	BS	plaiting her child		
4:20	ST	BS	plaiting her child		
4:25	TG		Opens the pot on the fire to check the yam	yam	
4:30	TG		Uses her hand to pick up a yam from the fire to test if the yam is soft	yam	
4:31	TG		Cover the pot on the fire		
4:32	TG		Bringing down the pot from the fire		

TIME	ACTOR INITIALS	RECIPIENT INITIALS	WHAT HAPPENED	FOOD	Reactivity
4:33	TG		Start removing the fire wood		
4:34	BS		Bringing different plate near the pot		
4:35	BS		Setting the plates		
4:36	TG		Start serving the yam into 3 plates		
4:37	TG		Serving the yam		
4:38	TG		Severs all the yam and cover the pot		
4:39	TG		Bring a bottle of palm oil	Palm oil and yam	
4:40	TG		Poured palm oil by the side of plate	Palm oil and yam	
4:45	BS		Start picking all the plate of yam to the kitchen	Palm oil and yam	
4:50	BS		Keep one plate of yam with 0il on the met	Palm oil and yam	
4:55	BS,TG		Washing their hand with just water	Palm oil and yam	
5:00	BS,TG		Start eating yam with palm oil together	Palm oil and yam	
5:05	BS,TG		Are eating together	Palm oil and yam	
5:10	SB		Playing all by himself		
5:15	BS,TG		Eating yam together	Palm oil and yam	
5:20	ST	SB	Start breast feeding her child	Breast milk	
5:25	ST	SB	breastfeeding	Breast milk	
5:30	ST	SB	breastfeeding	Breast milk	
5:35	ST	SB	breastfeeding	Breast milk	
5:40	ST	SB	breastfeeding	Breast milk	

5:45	ST	SB	Use a wrapper to piggyback her child on the back		
5:50	ST	SB	Carrying her child on the back		
5:55	ST	SB	Carrying her child on the back		
6:00	ST	SB	Carrying her child on the back		
6:05	ST		Lays the child in the room and cover the body with wrapper		
6:10	ST		Fetches water in a bucket		
6:15	ST		Goes into the bathroom with the bucket of water		
6:20	TG		Pours dry okro into the mortar	okro	
6:25	TG		Pounding dried okro	okro	
6:30	TG		Pounding dried okro	okro	
6:35	BS		Collects the pastel and also pounds	okro	
6:40	BS,TG		Pounding okro	okro	
6:45	BS,TG		Pounding okro	okro	
6:50	BS,TG		Goes into the kitchen to bring a spoon		
6:55	BS		Uses the spoon to fetch the dried okro into a small plate		
7:00	TG		Into the kitchen to cooks		

PARTICIPANT ID: __________

Primary Questions (Complete after you complete data collection):

From what you have seen observing THIS HOUSEHOLD, please answer the following questions in detail:

Question	Write your <u>detailed</u> response here
1. In this household, what would be the best way(s) to give nutritional supplements such as an MNP to young children to ensure compliance?<br><br>Why?	The best way to give MNP to the young child in this household is to give then in the morning. that is when the young child will give compliance.<br>Because during other time like in the afternoons or evening the young child reject any other food except the mothers breast milk .
2. In this household, who is favored during meal preparation or feeding?<br><br>How is this favoritism expressed?	The younger child is favored because all attention is on him when his semisolid food is ready (kunu)this because this younger child is presently ill.<br>It is expressed in such a way that his kunu is make separately from other children's kunu. And his kunu is always with groundnut pest.
3. How could we promote the MNP supplement for this family that would attract their attention?<br><br>Who would they listen to?	The MNP should be promoted through the mother because she understand the health condition of the child better than anyone else.<br>The would listen to the mother since she is always around

4. In this household, what would be the best ways to promote the MNP but limit how much it would be shared?<br><br>Why?	The best way to promote this product is still through the mother and the possibility that the product would be shared is limited because other children think the product is only meant for the younger child because he is ill.<br>The product may likely not capture the attention of other children because of the child condition the mother herself may not share the product because she want her child to uses all of it and get better
5. What other issues and concerns did you see with this family that are important for us to consider with regard to introduction of an MNP?<br><br>Why?	There are no other concerns as far as this household is concern only that the younger child present conditions is pathetic<br><br>The mother maintains a proper hygiene but due to the health status of this young child the mother may not be able to notice any impact the MNP has on the child yet.
6. Based on your experience observing this child today, do	There were two or three occasion when the mother would do

you believe reactivity negatively impacted your ability to see the food-related behaviors of the child?<br><br>Please explain your answer in detail.	something and check to if am watching her .aside from that my observation was no affected<br><br>The mother was trying to show that she is mindful of the young child hygiene by rinsing the spoon to feed the child with and washing her hand before preparing kunun.
